# Supplementary material for: Causal Relationship between Aging and Anorexia Nervosa: A White-Matter-Microstructure-Mediated Mendelian Randomization Analysis
Source: Biomedicines. 2024 Aug 16;12(8):1874. doi: 10.3390/biomedicines12081874 (PMC11351342; doi:10.3390/biomedicines12081874)
Supplement: Supplementary file 1 [file biomedicines-12-01874-s001.zip › Supplementary Figure/Figure S4/Singe SNP plot of MR analysis for TL to ubm-b-1557.pdf]

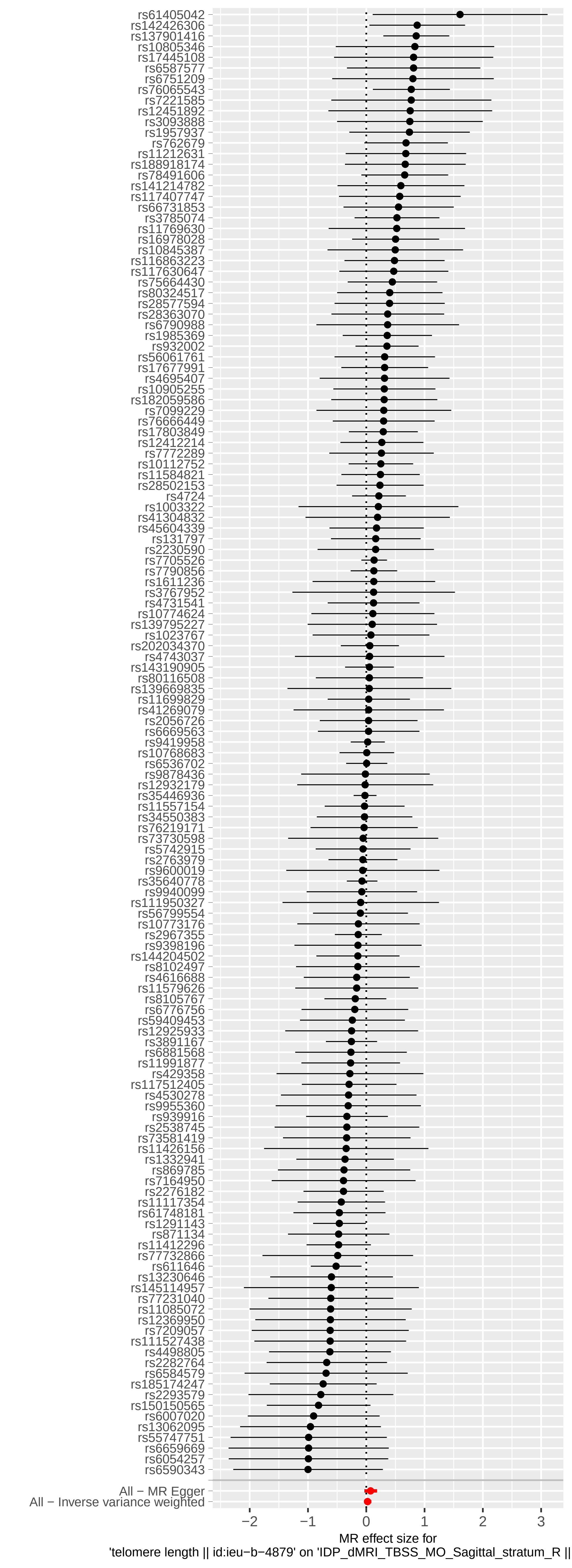
rs61405042
rs142426306
rs137901416
rs10805346
rs17445108
rs6587577
rs6751209
rs76065543
rs7221585
rs12451892
rs3093888
rs1957937
rs762679
rs11212631
rs188918174
rs78491606
rs141214782
rs117407747
rs66731853
rs3785074
rs11769630
rs16978028
rs10845387
rs116863223
rs117630647
rs75664430
rs80324517
rs28577594
rs28363070
rs6790988
rs1985369
rs932002
rs56061761
rs17677991
rs4695407
rs10905255
rs182059586
rs7099229
rs76666449
rs17803849
rs12412214
rs7772289
rs10112752
rs11584821
rs28502153
rs4724
rs1003322
rs41304832
rs45604339
rs131797
rs2230590
rs7705526
rs7790856
rs1611236
rs3767952
rs4731541
rs10774624
rs139795227
rs1023767
rs202034370
rs4743037
rs143190905
rs80116508
rs139669835
rs11699829
rs41269079
rs2056726
rs6669563
rs9419958
rs10768683
rs6536702
rs9878436
rs12932179
rs35446936
rs11557154
rs34550383
rs76219171
rs73730598
rs5742915
rs2763979
rs9600019
rs35640778
rs9940099
rs111950327
rs56799554
rs10773176
rs2967355
rs9398196
rs144204502
rs8102497
rs4616688
rs11579626
rs8105767
rs6776756
rs59409453
rs12925933
rs3891167
rs6881568
rs11991877
rs429358
rs117512405
rs4530278
rs9955360
rs939916
rs2538745
rs73581419
rs11426156
rs1332941
rs869785
rs7164950
rs2276182
rs11117354
rs61748181
rs1291143
rs871134
rs11412296
rs77732866
rs611646
rs13230646
rs145114957
rs77231040
rs11085072
rs12369950
rs7209057
rs111527438
rs4498805
rs2282764
rs6584579
rs185174247
rs2293579
rs150150565
rs6007020
rs13062095
rs55747751
rs6659669
rs6054257
rs6590343
All − MR Egger
All − Inverse variance weighted
−2
−1
0
1
2
3
MR effect size for
'telomere length || id:ieu−b−4879' on 'IDP_dMRI_TBSS_MO_Sagittal_stratum_R ||
